# Online learning performance and engagement during the COVID-19 pandemic: Application of the dual-continua model of mental health

**DOI:** 10.3389/fpsyg.2022.932777

**Published:** 2022-07-22

**Authors:** Jinwon Kim, Kibum Moon, Jiye Lee, Yaewon Jeong, Seungjin Lee, Young-gun Ko

**Affiliations:** ^1^Office of Digital Information, Korea University, Seoul, South Korea; ^2^School of Psychology, Korea University, Seoul, South Korea

**Keywords:** dual-continua model, mental well-being, mental disorder, online learning, learning management system, student engagement, academic achievement, academic distress

## Abstract

The COVID-19 pandemic has led to an abrupt transition from face-to-face learning to online learning, which has also affected the mental health of college students. In this study, we examined the relationship between students’ adjustment to online learning and their mental health by using the Dual-Continua Model. The model assumes that mental disorder and mental well-being are related yet distinct factors of mental health. For this purpose, 2,933 college students completed an online survey around the beginning of the Fall semester of 2020 (*N* = 1,724) and the Spring semester of 2021 (*N* = 1,209). We assessed participants’ mental well-being, mental disorders, and academic distress by means of the online survey. In addition, we incorporated grades and log data accumulated in the Learning Management System (LMS) as objective learning indicators of academic achievement and engagement in online learning. Results revealed that two dimensions of mental health (i.e., mental well-being and mental disorder) were independently associated with all objective and subjective online learning indicators. Specifically, languishing (i.e., low levels of mental well-being) was negatively associated with student engagement derived from LMS log data and academic achievement and was positively associated with self-reported academic distress even after we controlled for the effects of mental disorder. In addition, mental disorder was negatively related to student engagement and academic achievement and was positively related to academic distress even after we controlled for the effects of mental well-being. These results remained notable even when we controlled for the effects of sociodemographic variables. Our findings imply that applying the Dual-Continua Model contributes to a better understanding of the relationship between college students’ mental health and their adaptation to online learning. We suggest that it is imperative to implement university-wide interventions that promote mental well-being and alleviate psychological symptoms for students’ successful adjustment to online learning.

## Introduction

The COVID-19 pandemic has affected the entire world, and higher education is not an exception. The virus has unprecedentedly disrupted the educational system at all levels, affecting the lives of students of all ages because of preventive policies, such as on social distancing and lockdown (Raza et al., 2020). Facing the crisis, rapid and complete transitions to online learning have become a requisite not only for educational institutes and instructors but also for the students (Hasan and Bao, 2020). In South Korea, universities have conducted most classes online in following the guideline of the Ministry of Education since April 2020 (Ministry of Education, 2021). These dramatic changes have significantly affected the learning experiences and mental well-being of college students (Ihm et al., 2021).

As the primary focus of higher education shifts from face-to-face learning to online learning, the importance of a Learning Management System (LMS) has increased significantly (Raza et al., 2020). LMS refers to web-based technology or software applications that are intended to facilitate online learning processes (Alias and Zainuddin, 2005). An LMS is generally used to create and deliver learning content, keep track of student participation, and provide a platform for online interactions, such as threaded discussions or video conferences (Lonn et al., 2011; Emelyanova and Voronina, 2014). In an online learning environment based on LMS, diverse aspects of students’ learning activities can be automatically recorded in log files, which provide abundant information for analyzing learning processes and patterns of students (Black et al., 2008; Henrie et al., 2018). With the development of learning analytics with machine learning and deep learning techniques, emerging research suggests that LMS log data can be effectively used to predict academic performance (Hu et al., 2014; Costa et al., 2017; Dascalu et al., 2021; Moon et al., 2021; Riestra-González et al., 2021; Zhao et al., 2021); identify students with poor mental health (Heo et al., 2019); and provide academic and psychological interventions (Conijn et al., 2017; Cromley et al., 2020).

In addition, there is a growing interest in analyzing LMS log data to measure student engagement in online learning environments, especially from a behavioral perspective (Umer et al., 2018; Wong and Chong, 2018; Motz et al., 2019; Lu and Cutumisu, 2022). According to the theoretical framework of student engagement proposed by Fredricks et al. (2004), student engagement can be conceptualized in three dimensions: behavioral, cognitive, and emotional. Behavioral engagement refers to students’ involvement and persistence in learning activities. Cognitive engagement refers to students’ cognitive investment in learning complex ideas or difficult skills. Emotional engagement is defined as emotional reactions toward learning activities, teachers, peers, and academics. Compared to emotional and cognitive engagement, behavioral engagement may be more easily and objectively measured through analyzing LMS activity logs (Wang, 2017). Motz et al. (2019) also showed that LMS log data can be a valid proxy measure of behavioral engagement. Since behavioral engagement is a crucial element for promoting academic achievement and preventing dropouts (Fredricks et al., 2004; Archambault et al., 2009; Wang and Holcombe, 2010; Wang and Eccles, 2012), LMS log data can provide important information in assisting students academically.

Together with the ground-breaking changes in the educational paradigm, the COVID-19 pandemic also affects the mental health in the student population. Deteriorations in students’ mental health, such as exacerbated psychological stress, anxiety, and depression, come across as a global phenomenon (for review, see Ihm et al., 2021). Specifically, according to a cross-sectional study conducted during the lockdown, university students in Bangladeshi reported COVID-19-related stress, anxiety, and depression, which had significant associations with psychosocial variables, including perception of physical symptoms, fear of infection, financial uncertainty, and lack of engagements in recreational activities (Khan et al., 2020). Tang et al. (2020) also examined the one-month prevalence of post-traumatic stress disorder (PTSD) and depressive symptoms among Chinese university students after the outbreak of COVID-19, identifying that fear of infection and living in severely affected areas contribute as main risk factors for development of COVID-19-induced mental health problems. In addition, difficulties in adapting to an unfamiliar online learning environment have been reported to intensify anxiety, depression, and academic distress of college students (Wang et al., 2020; Fawaz and Samaha, 2021; Horita et al., 2021).

The COVID-19 pandemic has not only aggravated mental illness but also reduced mental well-being (Gray et al., 2020; Satici et al., 2020; Copeland et al., 2021). The general population’s mental well-being, operationalized by positive affect, level of functioning, and positive relationships, significantly decreased after the prolonged lockdown period compared to the data collected in 2019 before the pandemic (Gray et al., 2020). Satici et al. (2020) found that intolerance of uncertainty during the pandemic predicts a significant decline in mental well-being, serially mediated by rumination and fear of COVID-19. Similarly, research with college student samples revealed that the overall mood, wellness behavior, and happiness of college students significantly decreased after the COVID-19 crisis (Office for National Statistics, 2020; Copeland et al., 2021). Lyons et al. (2020) also emphasized the importance of providing school-level interventions to promote the mental well-being of students because 68% of the medical student sample reported a significant decrease in mental well-being since the onset of the COVID-19 pandemic. Moreover, it has been shown that academic distress caused by a sudden transition to online learning is negatively associated with college students’ mental well-being (Barbayannis et al., 2022).

Whereas mental illness and mental well-being are often regarded as being opposite ends of a single bipolar continuum, an alternative model, named the Dual-Continua Model, considers mental illness and mental well-being as separate but correlated unipolar dimensions of mental health (Greenspoon and Saklofske, 2001; Keyes, 2002, 2005, 2007). According to this positive mental-health model, a person with a mental disorder may experience high levels of mental well-being, whereas a person without mental disorders may experience low levels of mental well-being. Ryff and Singer (2003) suggested that positive mental health is closely correlated with resilience, an individual’s ability to cope and accomplish growth in the face of life stressors and crisis. Poor mental well-being, on the other hand, serves as a risk factor for mental illness (Keyes et al., 2010; Wood and Joseph, 2010; Iasiello et al., 2019). According to a longitudinal study conducted by Keyes et al. (2010), participants with poor mental well-being were six times more likely to develop mental illness over the 10-year period, than were those who had maintained or improved their well-being at both time points.

Based on the Dual-Continua Model, individuals’ mental health status can be categorized by their levels of mental well-being and mental illness (Keyes, 2005; Suldo and Shaffer, 2008). For example, Keyes (2005) classified individuals into five mental-health groups: completely mentally healthy, moderately mentally healthy, pure languishing, pure mental illness, and mental illness and languishing. To elaborate, completely mentally healthy group is characterized by high levels of mental well-being (i.e., flourishing) and the absence of mental illness. Moderately mentally healthy group is characterized by moderate levels of mental well-being combined with the absence of mental illness. Pure languishing group is defined as low level of mental well-being (i.e., languishing) without any mental illness. Pure mental illness group is defined as moderate-to-high levels of mental well-being and the presence of mental illness. Mental illness and languishing group is characterized by the presence of languishing combined with the presence of mental illness. Several studies have identified differences between mental-health groups in psychosocial functioning, educational functioning, and physical health (Keyes, 2005, 2007; Suldo and Shaffer, 2008; Renshaw and Cohen, 2014). For instance, Suldo and Shaffer (2008) reported that adolescents with complete mental health were more adaptive in academic outcomes, social functioning, and physical health than their languishing peers without mental illness were. In addition, among students with high levels of clinical symptoms, students with high levels of mental well-being experienced better physical health, more social support, and fewer social problems than those with low levels of mental well-being did.

Previous research has examined the utility of the Dual-Continua Model in understanding the relationship between mental health and adjustment of college students (Eklund et al., 2010; Keyes et al., 2012; Renshaw and Cohen, 2014; Antaramian, 2015). For example, students with low levels of mental well-being showed less engagement in college experience and poorer academic achievement than did those with high levels of mental well-being, among college students without clinical symptoms (Antaramian, 2015). Understanding the association between online learning and mental health based on the Dual-Continua Model may contribute to contriving interventions and policies for students without mental well-being and for those with mental disorders. The view that the absence of mental illness guarantees mental health may overlook the importance of psychological interventions for students who show poor mental well-being without mental illness. Online learning environments may require greater intrinsic motivation and self-regulation from learners (Klingsieck et al., 2012; Pelikan et al., 2021). Students with low levels of mental well-being may experience more difficulties adapting to the sudden transition to online learning due to the pandemic, considering that poorer mental well-being has been associated with lower intrinsic motivation (Bailey and Phillips, 2016). In addition, increasing number of students experienced declines in their mental well-being because of the prolonged COVID-19 crisis (Grubic et al., 2020; Lyons et al., 2020). Therefore, special attention is also needed for college students with poor mental well-being to adapt to an online learning environment more successfully. However, not enough works have been done on this topic, especially in the current online learning environments triggered by the pandemic. Although previous research has shown that drastic shifting to online learning is related to poorer mental health of college students (Fawaz and Samaha, 2021; Fruehwirth et al., 2021), to the best of our knowledge, no empirical study has applied the Dual-Continua Model to examine how college students’ mental health is related to academic adjustment, including academic engagement, achievement, and distress in online learning environments during the pandemic. Given that two dimensions of mental health is crucial for academic success (Suldo and Shaffer, 2008; Antaramian, 2015), this study may provide useful implications to develop psychological interventions to promote college students’ adaptation to the new learning environment caused by the pandemic.

Thus, we aim to examine the utility of the Dual-Continua Model to understand the relationship between students’ mental health and their adaptation to online learning environments during the COVID-19 pandemic. Based on Keyes’ Dual-Continua Model (Keyes, 2002, 2005), we postulated that two dimensions of mental health (i.e., mental well-being and mental illness) would be independently associated with the adaptation to online learning. We incorporated grades and LMS log data as objective measures of academic achievement and engagement and self-reported academic distress as a subjective measure.

## Materials and methods

### Participants

We collected data from the Online Mental Health Surveys, which were conducted around the beginning of the Fall semester of 2020 and the Spring semester of 2021 during the COVID-19 pandemic conditions. In addition, grades and LMS log data stored in the university’s database were provided by the Office of Digital Information upon the informed consent from participating students. This study was approved by the Institutional Review Board (IRB).

Participants were combined from both time points. We chose the inclusion criteria for data of courses, students, and the LMS log before proceeding with data analyses. Specifically, since we were interested in the association between positive mental health and online learning, we excluded the data from the courses with pass/fail grades or with less than 1 h of study time per week (i.e., 1-credit courses), which were mainly offline-lab lectures. In addition, we included only participants who took at least one course that met our criteria and ended up with 2,933 participants for analysis. Although students were allowed to participate in both time points, and 338 (13.03%) out of 2,595 participants responded twice, for the convenience of analysis, each semester’s responses were considered as independent. A final analytic sample consisted of 1,724 college students in 2020 and 1,209 in 2021. Among the sample, 57.65% were female (*n* = 1,691), and the average age was 21.19 (*SD* = 2.23, range: 17–35).

### Measures

#### Online learning

##### Course grades

Grade point average (GPA) is widely used to measure academic achievement and is known as an objective measure with high internal reliability and validity (Richardson et al., 2012). We incorporated grades for each course to generate the indicator of academic performance in online learning. The grade consisted of nine levels: 4.5 (A+), 4.0 (A), 3.5 (B+), 3.0 (B), 2.5 (C+), 2.0 (C), 1.5 (D+), 1 (D), and 0 (F). Since participants took multiple courses per semester (*M* = 5.09, *SD* = 1.33, range = 1–9), we calculated the GPAs by averaging the grades that a student received from all courses in a semester (*M* = 3.88, *SD* = 0.59, range = 0–4.5).

##### LMS log data

The LMS log data contain detailed information about the learning activities of students along with the event time. We preprocessed and aggregated the LMS log data to objectively measure the engagement level of college students in online learning. Among the 10 different types of log data in the Blackboard Database, we incorporated only login-attempt and course-access data, which we postulated were most relevant to learning activities. Login-attempt logs were generated whenever a student accessed the LMS. Course-access logs were recorded whenever a student conducted any learning activities, including, but not limited to, opening course materials and notices, watching online content, taking quizzes, uploading assignments, and participating in forum discussions. Since the contents of learning activity differed greatly across courses, we counted only the number of events regardless of the activity types to enable scalability and generalizability of the analyses. In addition, we did not consider the amount of time spent on the LMS in our further analyses, because previous studies have suggested that there were null or weak associations between online learning properties and online time spent (Macfadyen and Dawson, 2010; Heo et al., 2019).

From raw data, we calculated three types of indicators of the engagement level in online learning: *Log-Count*, *Log-Entropy*, and *Access-Ratio*. Log-Count represents how actively a student participated in online learning and was computed by the average number of learning activities a certain student had made per lecture and per semester. Login-attempt was treated like a learning activity for a lecture, in the calculation of Log-Count and the other two indicators that come in below. The log transform of Log-Count was used for analyses because this variable was not normally distributed. Log-Entropy represents the consistency (i.e., the degree of variation) of learning activity across a semester and was computed by the Shannon entropy (Shannon, 1949): *S* = −∑*p*_*i*_*log*⁡*p*_*i*_, where *p*_*i*_ is the relative frequency of learning activities at the *i*th day of a semester. A higher Log-Entropy means that the distribution of learning activities is relatively uniform throughout a semester, and a lower Log-Entropy shows that the frequency of learning activities is concentrated on specific days (e.g., cramming for midterm and final exams). Last, Access-Rate refers to the steadiness of online learning participation across a semester, regardless of the absolute amount of learning activities. Access-Rate was measured by the proportions of days on which a student conducted a learning activity over the number of total days in a semester. Accordingly, an Access-Rate of 0.8 means that a student participated in online learning activity at least once during 80% of all days in a semester.

##### Academic distress

To assess academic distress experienced in online learning during the pandemic, we used four items from the Counseling Center Assessment of Psychological Symptoms-34 (CCAPS-34; Locke et al., 2012). The items measure how much academic distress individuals have experienced over the past 2 weeks (e.g., “I am unable to keep up with my schoolwork”). This scale is scored on a 5-point Likert scale ranging from 0 (*not at all like me*) to 4 (*extremely like me*). We used the Korean version of the CCAPS-34 (Kim et al., 2017). The internal consistency of this scale was 0.81.

#### Mental health

Based on Keyes’s Dual-Continua Model (Keyes, 2002, 2005), we measured two dimensions of mental health by means of two self-reported questionnaires: Mental Health Continuum-Short Form (MHC-SF) and Korean Mental Disorder Inventory (K-MDI). The MHC-SF was developed by Keyes (2002) to measure mental well-being and was validated in Korean by Lim et al. (2012). The MHC-SF includes three subscales of emotional well-being (items 1–3; e.g., “During the past month, how often did you feel happy?”), social well-being (items 4–8; e.g., “During the past month, how often did you feel that you had something important to contribute to society?”), and psychological well-being (items 9–14; e.g., “During the past month, how often did you feel that you had experiences that challenged you to grow and become a better person?”). Emotional well-being is related to the presence of positive affect and subjective satisfaction with life (Keyes, 2002). Psychological well-being refers to how well individuals function in their personal lives (e.g., self-acceptance, personal growth, and purpose in life; Ryff, 1989). Social well-being represents an individual’s subjective evaluation of social functioning (e.g., social acceptance, social contribution, and social integration; Keyes, 1998). This 14-item scale is scored on a 6-point Likert scale ranging from 0 (*never*) to 5 (*everyday*). Moreover, the MHC-SF employs the diagnostic criteria to classify individuals into three categories: Flourishing (mentally healthy), Moderately mentally healthy, and Languishing (mentally unhealthy) (Keyes et al., 2008). The internal consistency of the MHC-SF was 0.93.

In addition, we used the K-MDI to distinguish the presence or absence of mental disorders. The K-MDI was developed during a national sample survey on the mental health of Koreans (Lim et al., 2010). It consists of 14 items to measure the degree of discomfort caused by various clinical symptoms (e.g., “I often feel depressed or sad”) and the degree to which an individual has difficulties in work or relationships because of clinical symptoms. This scale is answered on a 5-point Likert scale, except for the last question, which is answered on a 4-point Likert scale. The internal consistency of the K-MDI was 0.84. It has been validated and widely used to measure the two factors of mental health by using the MHC-SF and the K-MDI (Kim and Ko, 2012; Baek et al., 2019).

Considering the two separate dimensions of mental well-being and disorder, we divided participants into four mental-health groups: (1) *Flourishing and Moderate* (characterized by the presence of mental well-being and the absence of mental illness), (2) *Pure Languishing* (characterized by the absence of both mental well-being and mental illness), (3) *Pure Mental Disorder* (characterized by the presence of both mental well-being and mental illness), and (4) *Mental Disorder and Languishing* (characterized by the presence of mental illness combined with languishing). Table 1 summarizes the subgroups of mental health, as suggested by the Dual-Continua Model.

**TABLE 1:** Diagnostic categories of mental health.

Mental disorder	Mental well-being			
	Languishing		Moderate to flourishing	
No	Pure languishing		Flourishing and moderate	
	MHC-SF	Low level on at least one item (emotional well-being) and low level on six or more items (psychological and social well-being)	MHC-SF	Flourishing: High level on at least one item (emotional well-being) and high level on six or more items (psychological and social well-being) Moderately mentally healthy: Not in a state of flourishing or languishing
	K-MDI	A state that does not meet the criteria for diagnosis of mental disorders in K-MDI	K-MDI	A state that does not meet the criteria for diagnosis of mental disorders in K-MDI
Yes	Mental disorder and languishing		Pure mental disorder	
	MHC-SF	Low level on at least one item (emotional well-being) and low level on six or more items (psychological and social well-being)	MHC-SF	Flourishing: High level on at least one item (emotional well-being) and high level on six or more items (psychological and social well-being) Moderately mentally healthy: Not in a state of flourishing or languishing
	K-MDI	High level on at least one item (discomfort with clinical symptoms) and high level on dysfunction in daily life because of the clinical symptoms	K-MDI	High level on at least one item (discomfort with clinical symptoms) and high level on dysfunction in daily life because of the clinical symptoms

MHC-SF, Mental Health Continuum-Short Form; K-MDI, Korean Mental Disorder Inventory. Participants were divided into four mental-health groups. In the dimension of mental well-being, flourishing and moderately mentally healthy categories were combined as one category [Adapted from Suldo and Shaffer’s (2008) study].

### Statistical analyses

All statistical analyses of this study were conducted with R (R Core Team, 2021). First of all, sociodemographic differences between four mental-health groups were analyzed using chi-squared tests and the analyses of variance (ANOVA). Additionally, we conducted ANOVA to examine group differences in online learning indicators (i.e., academic distress, GPA, Log-Count, Log-Entropy, and Access-Rate). Later, the relationships between online learning indicators were analyzed using Pearson’s correlation. For the main analysis, we then tested the association between two dimensions of mental health and online learning indicators by fitting linear regression models. We examined the hypotheses for online learning using linear mixed-effect models because students were allowed to participate in both time points, and 338 (13.03%) out of 2,595 participants responded twice. To overcome the problem of non-independence of observations, we tested linear mixed-effects models with a random intercept for each student and fixed slopes for mental well-being, mental disorder, and covariates. We used the lmer function in the lme4 package (Bates et al., 2015) to test linear mixed-effects models in this study. In addition, we used the Satterthwaite approximation for degrees of freedom to conduct significance testing (Kuznetsova et al., 2017).

## Results

The descriptive statistics for study variables by mental-health groups are presented in Table 2. The skewed distribution of Log-Count was log-transformed for further statistical analyses. We conducted chi-squared tests and ANOVA to examine the association between mental-health types and sociodemographic factors of participants. We found that the distribution of mental-health groups was significantly, but weakly, associated with sex (χ^2^ = 13.951, *p* = 0.003), age [*F*(3, 2929) = 8.891, *p* < 0.001, η^2^ = 0.009], annual household income [*F*(3, 2929) = 2.734, *p* < 0.05, η^2^ = 0.003], and applied credit per semester [*F*(3, 2929) = 3.336, *p* < 0.05, η^2^ = 0.003], not with data collection time point (χ^2^ = 3.454, *p* = 0.327) and school year (χ^2^ = 12.233, *p* = 0.201). All sociodemographic variables were used as covariates for the main analyses.

**TABLE 2:** Descriptive statistics for study variables by mental-health groups (*N* = 2,933).

Variable	Flourishing and moderate (*n* = 2276, 77.60%)		Pure languishing (*n* = 454, 15.48%)		Pure mental disorder (*n* = 125, 4.26%)		Mental disorder and languishing (*n* = 78, 2.66%)	
	% (*n*)	*Mean (SD)*	% (*n*)	*Mean (SD)*	% (*n*)	*Mean (SD)*	% (*n*)	*Mean (SD)*
Mental well-being^a^		50.48 (11.03)		29.07 (5.29)		45.50 (9.91)		25.47 (5.37)
Mental disorder^b^		21.61 (5.85)		26.36 (6.71)		33.74 (7.69)		40.00 (6.23)
Academic distress		4.34 (3.23)		7.98 (3.56)		6.92 (3.51)		11.59 (3.32)
GPA		3.92 (0.54)		3.83 (0.64)		3.64 (0.69)		3.35 (1.07)
Log-Count^c^		422.97 (199.96)		379.93 (186.99)		356.44 (168.53)		368.02 (179.45)
Log-Entropy		3.52 (0.22)		3.48 (0.25)		3.41 (0.33)		3.36 (0.37)
Access-Rate		0.51 (0.09)		0.49 (0.09)		0.47 (0.10)		0.46 (0.11)
*Covariates*								
Year—Term								
2020—Fall	77.96		15.78		3.71		2.55	
2021—Spring	77.09		15.05		5.05		2.81	
Sex								
Female (%)	75.58		17.45		4.02		2.96	
Male (%)	80.35		12.80		4.59		2.25	
School Year (%)								
1	81.02		13.09		3.80		2.09	
2	78.48		14.92		3.87		2.73	
3	76.96		15.85		4.26		2.93	
4	73.79		18.17		5.13		2.91	
Age (year; range: 17–35)		21.09 (2.18)		21.38 (2.31)		21.85 (2.42)		21.88 (2.46)
Annual Household Income (range: 1–10)^d^		6.60 (2.85)		6.19 (2.91)		6.38 (3.09)		6.56 (3.10)
Applied Credit		15.01 (3.77)		14.52 (4.08)		14.57 (4.14)		14.18 (4.06)

^a^The mean of all participants for mental well-being was 46.29 (SD = 13.22).

^b^The mean of all participants for mental disorder was 23.35 (SD = 7.26).

^c^The means and standard deviations of Log-Count were calculated with raw data before log transformation.

^d^Annual Household Income is divided equally by 10% based on income, with the lowest income level (lower 10%) as the 1st decile and the highest level (top 10%) as the 10th decile. We treated this variable as continuous.

In addition, we conducted ANOVA to examine the differences in online learning indicators between four mental health groups. The results showed significant main effects of mental health groups on all five online learning indicators: academic distress [*F*(3, 2929) = 267.867, *p* < 0.001, η^2^ = 0.215], GPA [*F*(3, 2929) = 32.987, *p* < 0.001, η^2^ = 0.033], Log-Count [*F*(3, 2929) = 19.785, *p* < 0.001, η^2^ = 0.020], Log-Entropy [*F*(3, 2929) = 21.564, *p* < 0.001, η^2^ = 0.022], and Access-Rate [*F*(3, 2929) = 22.387, *p* < 0.001, η^2^ = 0.022]. *Post-hoc* analyses with Bonferroni corrections indicated every group showed significant differences in academic distress. Academic distress for each group was shown to be high in order of Mental Disorder and Languishing, Pure Languishing, Pure Mental Disorder, and Flourishing and Moderate (*p* < 0.01). We also found that every group showed significant differences in the GPA. The average GPAs for each group were shown to be high in the order of Flourishing and Moderate, Pure Languishing, Pure Mental Disorder, and Mental Disorder and Languishing (*p* < 0.05). As for the engagement indicators (i.e., Log-Count, Log-Entropy, and Access-Rate), the means of the Flourishing and Moderate group were the highest in all models (*p* < 0.01). Apart from the most adaptive group, the other three mental health groups did not significantly differ in terms of Log-Count. However, participants classified as Pure Languishing group showed higher levels of Log-Entropy (*p* < 0.001) and Access-Rate (*p* < 0.05) than did those classified as Mental Disorder and Languishing group. In addition, Pure Languishing group showed higher levels of Log-Entropy than did Pure Mental Disorder group (*p* < 0.05).

The correlations between online learning variables are presented in Table 3. The Pearson’s correlation coefficients showed significant small-to-large correlations among objective online learning indicators (ranging from 0.22 to 0.85). In addition, self-reported academic distress was more significantly correlated with GPA than with other objective online learning indicators (*r* = -0.25, *p* < 0.001).

**TABLE 3:** Descriptive statistics and correlations between online learning indicators (*N* = 2,933).

	Academic Distress	GPA	Log-Count	Log-Entropy	Access-Rate
Academic distress	–				
GPA	–0.25	–			
Log-Count	–0.14	0.22	–		
Log-Entropy	–0.12	0.23	0.60	–	
Access-Rate	–0.16	0.24	0.79	0.85	–
*M*	5.21	3.88	412.02^a^	3.50	0.51
*SD*	3.72	0.59	197.25^a^	0.24	0.09

GPA, Grade Point Average. All correlations were significant (p < 0.001).

^a^The mean and standard deviation of Log-Count were calculated with raw data before log transformation.

We tested our main hypothesis of whether two factors of mental health (i.e., mental well-being and mental illness) would be independently associated with academic adjustment, including academic achievement, engagement, and distress in online learning environments. The summary of results is displayed in Table 4. The results revealed that mental well-being and mental disorder were independently associated with all five subjective and objective online learning indicators after we controlled for the effects of covariates. Specifically, in terms of mental well-being, languishing students showed poorer academic achievement, less engagement in online learning, and greater academic distress than did flourishing and moderately mentally healthy students. Furthermore, this pattern was also found in the dimension of mental disorder. Participants with mental illness showed more academic distress, less engagement, and poorer academic achievement than did those without mental illness.

**TABLE 4:** Results of the linear mixed-effect models in predicting online learning indicators (*N* = 2,933).

	Academic Distress		GPA		Log-Count		Log-Entropy		Access-Rate	
	β	95% CI	β	95% CI	β	95% CI	β	95% CI	β	95% CI
Fixed effects										
Languishing (reference: flourishing and moderately mentally healthy)	0.89***	0.81, 0.97	–0.18***	–0.27, –0.09	–0.14**	–0.23, –0.06	–0.11*	–0.21, –0.02	–0.15**	–0.23, –0.06
Mental Disorder (reference: without mental disorder)	0.72***	0.59, 0.84	–0.48***	–0.62, –0.34	–0.28***	–0.41, –0.15	–0.42***	–0.56, –0.28	–0.33***	–0.46, –0.19
Age	0.07**	0.02, 0.13	–0.19***	–0.25, –0.13	–0.02	–0.07, 0.04	–0.11***	–0.17, –0.05	–0.09**	–0.15, –0.03
School Year [2] (reference: school year [1])	0.02	–0.07, 0.11	0.05	–0.04, 0.15	–0.62***	–0.71, –0.52	–0.27***	–0.37, –0.17	–0.40***	–0.49, -0.30
School Year [3] (reference: school year [1])	–0.02	–0.14, 0.09	0.24***	0.12, 0.37	–0.62***	–0.74, –0.50	–0.10	–0.23, 0.02	–0.36***	–0.48, –0.24
School Year [4] (reference: school year [1])	–0.06	–0.20, 0.07	0.44***	0.29, 0.59	–0.75***	–0.89, –0.60	–0.17*	–0.32, –0.02	–0.47***	–0.62, –0.32
Sex (female; reference: male)	0.25***	0.18, 0.33	0.15***	0.07, 0.23	–0.11**	–0.18, –0.03	–0.20***	–0.29, –0.12	–0.31***	–0.39, –0.23
Annual Household Income	–0.10***	–0.14, –0.07	0.03	–0.00, 0.07	0.02	–0.01, 0.06	–0.01	–0.04, 0.03	0.03	–0.01, 0.06
Applied Credit	–0.01	–0.05, 0.02	0.06***	0.03, 0.10	0.21***	0.17, 0.24	–0.01	–0.04, 0.03	0.03	–0.01, 0.06
Year (2021; reference: 2020)	0.13***	0.07, 0.18	–0.10**	–0.17, –0.04	–0.10**	–0.16, –0.04	0.06	–0.00, 0.13	–0.12***	–0.18, –0.06
Random effects										
σ^2^	4.31		0.13		0.07		0.02		0.00	
τ_00_	6.26		0.21		0.09		0.03		0.00	
ICC	0.59		0.62		0.55		0.55		0.63	

CI, Confidence Interval; σ^2^,Within-Person Variance; τ_00_, Between-Person Variance; ICC, Intraclass Correlation Coefficient. All dependent and independent variables were standardized except for the categorical variables (i.e., two dimensions of mental health, school year, sex, and year).

*p < 0.05, **p < 0.01,***p < 0.001.

**FIGURE 1:**
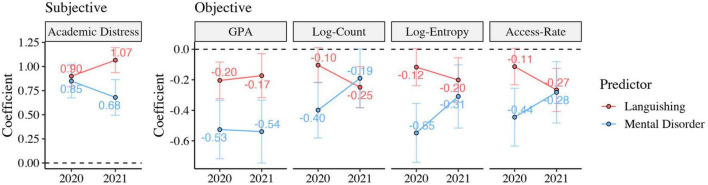
The differences in the effects of mental well-being and mental disorder on online learning indicators by the time point. The plot shows the multiple regression coefficients of two dimensions of mental health in predicting online learning indicators (for detailed statistics, see Supplementary Tables 1, 2). The error bar represents 95% confidence intervals. Sociodemographic variables were controlled as covariates in the multiple linear regression models. Red lines indicate the effects of languishing (reference: flourishing and moderately mentally healthy) on online learning variables. Blue lines indicate the effects of mental disorder (reference: without mental disorder) on online learning variables.

In addition, we explored whether the effects of mental well-being and mental disorder on online learning indicators differ by the time point. As shown in Figure 1, in 2020, mental disorder was independently related to all online learning indicators. Mental well-being was significantly associated with academic distress and GPA but was not significantly related to the other three indicators. In 2021, both dimensions of mental health were independently associated with all online learning indicators after we controlled for the effects of covariates, except for the relationship between mental disorder and Log-Count (for detailed statistics, see Supplementary Tables 1, 2).

## Discussion

Using the Dual-Continua Model (Keyes, 2002, 2005), we examined the effects of mental health on college students’ adaptation to online learning environments during the COVID-19 pandemic. We employed GPAs and LMS log data as objective indicators of academic achievement and engagement and used self-reported academic distress as a subjective indicator. In support of our hypothesis, we found that the two dimensions of mental health (i.e., mental well-being and mental disorder) were independently associated with college students’ engagement in online learning. Specifically, the two dimensions were significantly associated with how actively and consistently students engaged in online learning activities. Furthermore, each factor of mental health was independently related to academic achievement and self-reported academic distress. These findings remained significant after controlling for the effects of sociodemographic variables.

In terms of mental well-being, we demonstrated that participants classified as flourishing and moderately healthy took part in online classes more actively and consistently and obtained higher GPAs than did those classified as languishing. This is consistent with previous literature that students with higher levels of mental well-being were more likely to show higher engagement in class (Lewis et al., 2011; Montano, 2021) and higher academic achievement (Howell, 2009) than those with lower levels of mental well-being. Our findings highlighted that individuals’ mental well-being played an important role in their adaptation to online learning environments during the pandemic. Student well-being is a critical factor for maintaining learning motivation (Yang et al., 2021). Past research has suggested that intrinsic academic motivation may also contribute to successful academic engagement or adaptive learning (Elphinstone and Farrugia, 2016). Given that online contexts require greater self-regulation and intrinsic motivation from the learners (Klingsieck et al., 2012; Pelikan et al., 2021), an abrupt transition to online learning during the pandemic could have obstructed languishing students from adapting to online learning.

We also found that participants with mental illness showed higher levels of academic distress, poorer academic performance, and lower levels of engagement in online learning than did those without mental illness. These findings are in accordance with previous studies that college students with mental disorders were likely to experience difficulties adapting to their campus life and classes (e.g., Salzer, 2012). Moreover, the pandemic led to a dramatic change from face-to-face learning to online learning. Maladjustment to online courses caused by rapid changes in the educational paradigm has been closely related to psychological difficulties, such as anxiety and depression (Fawaz and Samaha, 2021; Fruehwirth et al., 2021).

Our findings that two factors of mental health were independently associated with adjustment to online learning provide additional evidence that mental well-being and mental illness are interrelated but distinct constructs. Consistent with a study conducted by Suldo and Shaffer (2008), we suggest that it is necessary to foster college students’ mental well-being because the absence of mental illness is not sufficient for them to successfully adapt to online learning environments. Our data also illustrated that Pure Languishing group (i.e., low levels of mental well-being without any mental illness) showed lower mental well-being and perceived greater academic distress than did Pure Mental Disorder group (i.e., average to high mental well-being and the presence of mental disorder). This may imply that it could be helpful to encourage Pure Languishing group to actively participate in mental well-being promotion programs. Moreover, for students with mental disorders, we propose that it may be important to establish interventions with different strategies depending on their level of mental well-being. For example, interventions focused on alleviating clinical symptoms can be effective for Pure Mental Disorder group. On the other hand, for students in Mental Disorder and Languishing group, it may be important that universities focus not only on reducing their clinical symptoms but also on improving their mental well-being for their academic success. In addition, online learning requires greater intrinsic motivation for students to regulate their learning on their own (Klingsieck et al., 2012; Pelikan et al., 2021). Given that higher mental well-being is related to higher intrinsic motivation (Bailey and Phillips, 2016), psychological interventions that encompass the two factors of mental health can lead to optimal academic functioning. Over the past years, the importance of online learning has increased, and online learning environments are expected to continue after the pandemic. In this situation, our study is of academic significance in that we identified the utility of the Dual-Continua Model of mental health to understand the relationship between mental health and the adaptation to online learning triggered by the pandemic. Taken together, we propose that it is essential to establish university-level interventions based on positive psychology for their successful adaptation to an online learning environment.

Furthermore, we suggest that the application of LMS log data has notable advantages in measuring student engagement. Since students’ learning activities are automatically recorded in log files (Black et al., 2008), an analysis of LMS log data can show how students engage in online learning environments. Student engagement has been mainly measured by participants’ perceptions of their engagement in learning activities (Chen et al., 2010; Klobas and McGill, 2010; Junco et al., 2011). Unlike self-reports, which can be affected by social desirability or impression management (Paulhus, 1984), LMS logs can be used to objectively measure students’ actual engagement. Motz et al. (2019) suggested that LMS log data can be a useful and valid measure of student engagement by demonstrating that instructors’ subjective ratings of engagement were closely associated with student engagement derived from LMS log data. Our data also revealed that the three types of indicators of student engagement (i.e., Log-Count, Log-Entropy, and Access-Rate) were positively correlated with academic performance and negatively with self-reported academic distress. Moreover, we propose a novel approach to extract indicators of student engagement showing students’ learning patterns from massive LMS log data. Log-Entropy and Access-Rate represent how consistently students participate in online learning activities. These indicators may help distinguish students who consistently engage in learning activities throughout a semester from those who only engage in learning activities more intensively on specific days (e.g., cramming for exams), among students with high frequencies of activities. As the role and importance of online learning has increased, an analysis of LMS log data can provide meaningful insights into understanding students’ adaptation to online learning.

### Limitations

There are a few limitations of this study that should be noted. First, we used features derived from LMS log data to measure behavioral engagement. However, it may be necessary to consider a holistic approach to measure student engagement. Thus, future studies could comprehend the relationship between two factors of mental health and all three types of engagement (i.e., behavioral, emotional, and cognitive engagement). Second, we did not control for differences in characteristics between online classes or departments. For example, college students who take lectures requiring few assignments or lectures without active discussion in the LMS may use the LMS less frequently regardless of their mental health. Therefore, future research is needed to examine whether the two factors of mental health are independently related to the adaptation to online learning under the same class or departmental conditions. Finally, we assessed participants’ mental health using only self-reported measures. Although self-reports have been widely used to measure mental well-being and mental illness (e.g., Keyes et al., 2008), they may be affected by social desirability and self-deception. Further studies can employ clinician assessments to evaluate individuals’ mental well-being and mental illness more reliably and classify mental-health types based on the Dual-Continua Model.

## Conclusion

Overall, our results provide additional evidence that mental well-being and mental disorder are interrelated yet distinct dimensions of mental health. Using the Dual-Continua Model of mental health, we found that the two dimensions of mental health were independently associated with student engagement, academic achievement, and academic distress in an online learning environment. The effect of each factor of mental health on online learning indicators remained significant even after we controlled for the effects of sociodemographic variables. We suggest that it may be necessary to carry out university-wide interventions to promote mental well-being and alleviate clinical symptoms for college students to adapt to online learning environments more successfully. A remaining issue for future studies is whether the Dual-Continua Model of mental health can be applied to understanding the relationship between mental health and adjustment to online learning in the post-COVID-19 era.

## Data availability statement

The raw data supporting the conclusions of this article will be made available by the authors, without undue reservation.

## Ethics statement

This study was approved by the Institutional Review Board at Korea University (KUIRB-2021-0070-01 and KUIRB-2021-0126-01). The patients/participants provided their written informed consent to participate in this study.

## Author contributions

JK, KM, SL, and YK conceived and designed the study. JK, KM, and SL collected the data. JK and KM performed the data analysis and interpretation under the supervision of YK. JK, KM, JL, and YJ drafted the manuscript. KM and YK provided critical revisions. All authors read and approved the final version of the manuscript.

## Conflict of interest

The authors declare that the research was conducted in the absence of any commercial or financial relationships that could be construed as a potential conflict of interest.

## Publisher’s note

All claims expressed in this article are solely those of the authors and do not necessarily represent those of their affiliated organizations, or those of the publisher, the editors and the reviewers. Any product that may be evaluated in this article, or claim that may be made by its manufacturer, is not guaranteed or endorsed by the publisher.
