# Supplementary material for: Online learning performance and engagement during the COVID-19 pandemic: Application of the dual-continua model of mental health
Source: Front Psychol. 2022 Jul 22;13:932777. doi: 10.3389/fpsyg.2022.932777 (PMC9356232; doi:10.3389/fpsyg.2022.932777)
Supplement: Supplementary file 1 [file Data_Sheet_1.docx]

Supplementary Material

|  | **Academic Distress** | | **GPA** | | **Log-Count** | | **Log-Entropy** | | **Access-Rate** | |
| --- | --- | --- | --- | --- | --- | --- | --- | --- | --- | --- |
|  | β | 95% CI | β | 95% CI | β | 95% CI | β | 95% CI | β | 95% CI |
| Languishing (reference: flourishing and moderately mentally healthy) | 0.90^***^ | 0.79,  1.01 | –0.20^***^ | –0.32,  –0.08 | ­–0.10 | –0.22,  0.01 | –0.12 | –0.24,  0.00 | –0.11 | –0.23,  0.01 |
| Mental Disorder (reference: without mental disorder) | 0.85^***^ | 0.67,  1.02 | –0.53^***^ | –0.72,   –0.33 | –0.40^***^ | –0.58,   –0.22 | –0.55^***^ | –0.74,  –0.36 | –0.44^***^ | –0.63,  –0.26 |
| Age | 0.09^**^ | 0.02,  0.17 | –0.22^***^ | –0.30,  –0.14 | –0.04 | –0.12,  0.03 | –0.16^***^ | –0.24,  –0.08 | –0.11^**^ | –0.19,  –0.03 |
| School Year [2] (reference: school year [1]) | 0.09 | –0.04,  0.21 | 0.04 | –0.11,  0.18 | –0.47^***^ | –0.60,  –0.33 | –0.17^*^ | –0.31,  –0.03 | –0.27^***^ | –0.41,  –0.13 |
| School Year [3] (reference: school year [1]) | –0.02 | –0.17,  0.13 | 0.18^*^ | 0.02,  0.35 | –0.50^***^ | –0.66,  –0.35 | 0.01 | –0.15,  0.18 | –0.25^**^ | –0.41,  –0.09 |
| School Year [4] (reference: school year [1]) | –0.04 | –0.22,  0.14 | 0.48^***^ | 0.28,  0.68 | –0.59^***^ | –0.78,  –0.40 | –0.03 | –0.23,  0.17 | –0.39^***^ | –0.59,  –0.19 |
| Sex (female; reference: male) | 0.30^***^ | 0.21,  0.39 | 0.08 | –0.02,  0.19 | –0.12^*^ | –0.22,  –0.02 | –0.17^**^ | –0.27,  –0.07 | –0.28^***^ | –0.39,  –0.18 |
| Annual Household Income | –0.10^***^ | –0.14,  –0.05 | 0.04 | –0.01,  0.08 | 0.03 | –0.01,  0.07 | –0.02 | –0.06,  0.03 | 0.02 | –0.03,  0.06 |
| Applied Credit | –0.02 | –0.06,  0.03 | 0.10^***^ | 0.05,  0.15 | 0.21^***^ | 0.16,  0.26 | 0.02 | –0.03,  0.07 | 0.04 | –0.01,  0.09 |
| *Note*. All dependent and independent variables were standardized except for the categorical variables (i.e., two dimensions of mental health, school year, and sex).  ^*^ *p* < .05, ^**^ *p* <. 01, ^***^ *p* < .001 | | | | | | | | | | |

# Supplementary Table S1. Results of the multiple linear regressions in predicting online learning indicators in 2020 (*N* = 1,724)

|  | **Academic Distress** | | **GPA** | | **Log-Count** | | **Log-Entropy** | | **Access-Rate** | |
| --- | --- | --- | --- | --- | --- | --- | --- | --- | --- | --- |
|  | β | 95% CI | β | 95% CI | β | 95% CI | β | 95% CI | β | 95% CI |
| Languishing (reference: flourishing and moderately mentally healthy) | 1.07^***^ | 0.94,  1.20 | –0.17^*^ | –0.32,  –0.03 | ­–0.25^***^ | –0.38,  –0.11 | –0.20^**^ | –0.35,  –0.06 | –0.27^***^ | –0.41,  –0.13 |
| Mental Disorder (reference: without mental disorder) | 0.68^***^ | 0.49,  0.86 | –0.54^***^ | –0.74,   –0.33 | –0.19 | –0.38,   0.00 | –0.31^**^ | –0.52,  –0.10 | –0.28^**^ | –0.48,  –0.08 |
| Age | 0.08 | –0.00,  0.16 | –0.20^***^ | –0.29,  –0.11 | –0.00 | –0.09,  0.08 | –0.06 | –0.15,  0.03 | –0.07 | –0.16,  0.01 |
| School Year [2] (reference: school year [1]) | –0.08 | –0.23,  0.08 | 0.18^*^ | 0.01,  0.35 | –0.90^***^ | –1.06,  –0.74 | –0.41^***^ | –0.58,  –0.24 | –0.62^***^ | –0.79,  –0.46 |
| School Year [3] (reference: school year [1]) | –0.14 | –0.32,  0.04 | 0.43^***^ | 0.23,  0.63 | –0.77^***^ | –0.95,  –0.58 | –0.17 | –0.37,  0.03 | –0.39^***^ | –0.58,  –0.19 |
| School Year [4] (reference: school year [1]) | –0.19 | –0.40,  0.02 | 0.61^***^ | 0.38,  0.85 | –0.94^***^ | –1.16,  –0.72 | –0.26^*^ | –0.50,  –0.03 | –0.51^***^ | –0.74,  –0.28 |
| Sex (female; reference: male) | 0.20^***^ | 0.09,  0.31 | 0.20^**^ | 0.08,  0.32 | –0.11 | –0.23,  0.00 | –0.27^***^ | –0.39,  –0.15 | –0.35^***^ | –0.47,  –0.23 |
| Annual Household Income | –0.11^***^ | –0.16,  –0.06 | 0.02 | –0.03,  0.08 | 0.02 | –0.03,  0.07 | 0.02 | –0.04,  0.07 | 0.05 | –0.01,  0.10 |
| Applied Credit | 0.01 | –0.04,  0.07 | 0.06 | –0.00,  0.12 | 0.22^***^ | 0.16,  0.27 | –0.03 | –0.10,  0.03 | 0.03 | –0.03,  0.09 |
| *Note*. All dependent and independent variables were standardized except for the categorical variables (i.e., two dimensions of mental health, school year, and sex).  ^*^ *p* < .05, ^**^ *p* <. 01, ^***^ *p* < .001 | | | | | | | | | | |

# Supplementary Table S2. Results of the multiple linear regressions in predicting online learning indicators in 2021 (*N* = 1,209)
